# Predictive value of the THRIVE score for outcome in patients with acute basilar artery occlusion treated with thrombectomy

**DOI:** 10.1002/brb3.1418

**Published:** 2019-09-26

**Authors:** Beilei Chen, Liu Yang, Jing Hang, Shoujiang You, Jun Li, Xiaobo Li, Liangzhu Wang, Li Jiang, Wei Li, Hailong Yu

**Affiliations:** ^1^ Clinical Medical College of Yangzhou University Yangzhou City China; ^2^ Department of Neurology Northern Jiangsu People's Hospital Yangzhou City China; ^3^ Dalian Medical University Dalian City China; ^4^ Department of Neurology and Suzhou Clinical Research Center of Neurological Disease The Second Affiliated Hospital of Soochow University Suzhou City China; ^5^ Drum Tower Hospital Medical School of Nanjing University Nanjing City China

**Keywords:** basilar artery occlusion, endovascular treatment, stroke, THRIVE score

## Abstract

**Background and purpose:**

A higher Totaled Health Risks in Vascular Events (THRIVE) score has been shown to predict poor functional outcome in patients with acute ischemic stroke (AIS) and anterior circulation large vessel occlusions undergoing thrombectomy treatment. We attempted to evaluate the value of the THRIVE score in predicting the outcome of thrombectomy treatment in AIS patients with basilar artery occlusion (BAO).

**Methods:**

A total of 68 AIS patients with BAO who underwent thrombectomy treatment from May 2014 to August 2018 were included in the present study. Multivariable logistic regression was performed to determine the predictive value of the THRIVE score for poor functional outcome (defined as modified Rankin Scale score ≥ 3), all‐cause mortality, and hemorrhage transformation (HT) at 3 months.

**Results:**

A total of 42 (61.8%) participants experienced poor functional outcomes, 25 (36.8%) patients died from all causes, and 21 (30.9%) patients had HT during the 3‐month follow‐up. Multivariable logistic regression showed that a higher THRIVE score was significantly associated with poor functional outcome (odds ratio [OR] 5.86, 95% confidence interval [CI], 2.28–14.91, *p* < .001) as well as all‐cause mortality (OR 2.40, 95% CI, 1.32–4.34, *p* = .004) but not HT (*p* = .607). The C‐statistic of the THRIVE score was significantly larger than that of the NIHSS score for predicting poor functional outcome (AUC = 0.913; cutoff > 5; sensitivity, 88.5%; specificity, 83.3%, *p* = .007) and all‐cause mortality (AUC = 0.768; cutoff > 5; sensitivity, 92.0%; specificity, 65.1%, *p* = .018).

**Conclusions:**

A high THRIVE score was independently associated with an increased risk of poor functional outcome and all‐cause mortality in AIS patients with BAO who underwent thrombectomy treatment. Moreover, the THRIVE score appeared to be a better predictor of clinical outcome than the NIHSS score.

## Graphical abstract

A high THRIVE score was independently associated with an increased risk of poor functional outcomes and all‐cause mortality in AIS patients with basilar artery occlusion who underwent thrombectomy treatment. Moreover, the THRIVE score appeared to be a better predictor of clinical outcome than NIHSS score.

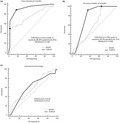

## 1 INTRODUCTION

Early recanalization is imperative for the treatment of patients with occlusion of large blood vessels. However, intravenous thrombolysis alone is limited to dissolving large blood clots. Mechanical thrombectomy represents the most effective treatment for the occlusion of large blood vessels (Ciccone et al., 2013). The effectiveness of endovascular stent thrombectomy (EST) is widely recognized by several very important clinical studies (MR CLEAN, ESCAPE, SWIFT PRIME, EXTEND‐IA, and REVASCAT; Campbell et al., 2014; Demchuk et al., 2015; Fransen et al., 2014; Molina et al., 2015; Saver et al., 2015). Based on recent studies regarding meta‐analyses of these five randomized clinical trials, EST is believed to be superior to medical therapy in improving outcome in patients with anterior circulatory vessel occlusion (Badhiwala et al., 2015; Bush et al., 2016). The Totaled Health Risks in Vascular Events (THRIVE) score was developed with data from the MERCI and Multi‐MERCI trials. The THRIVE score combines a patient's functional assessment with risk factors such as old age, hypertension, and hyperglycemia, which are closely related to the prognosis of ischemic stroke (Nakayama, Jorgensen, & Raaschou, 1994; Steger et al., 2004). This score is believed to be a good predictor of outcome after thrombectomy with the use of stents in patients with anterior circulatory vessel occlusion (Flint, Cullen, & Faigeles, 2010; Flint et al., 2013). Unfortunately, even though patients with basilar artery occlusion (BAO) have achieved good recanalization, the mortality rate at 90 days is still more than 35% (Fahed et al., 2017). Thrombectomy in posterior circulation strokes has not been studied extensively due to the lack of randomized trials (Gory et al., 2015; Kumar & Shahripour, 2014), and predictors of outcome in posterior circulation stroke are not understood. Whether the THRIVE score is effective in predicting the prognosis of patients with BAO after EST is still unclear. Therefore, our study evaluated the predictive value of the THRIVE score in acute ischemic stroke (AIS) patients with BAO who underwent EST.

## 2 METHODS

### 2.1 Study participants

From May 2014 to August 2018, we prospectively identified AIS patients with BAO who underwent EST treatment in Northern Jiangsu People's Hospital. Patients were enrolled if they met the following criteria: (a) patient was ≥18 years old; (b) patient had BAO diagnosed with CTA or MRA before EST; (c) patient underwent EST within 12 hr after symptom onset; (d) patient had a modified Rankin score (mRS) ≤ 1 before stroke; (e) patient underwent EST with Solitaire AB or combined with additional balloon and/or stenting angioplasty; and (f) patient received good revascularization. The exclusion criteria were as follows: (a) history of surgery or trauma within 2 months; (b) intracranial hemorrhage or history of subarachnoid hemorrhage, tumor, intracranial hemorrhage, or arteriovenous malformation; (c) large infarct core (exceeding two‐thirds of the midbrain, pons, or either side of the cerebellum); (d) dysfunction of important organs; (e) a definite bleeding tendency; (f) contrast or an aesthetic allergy; or (g) SBP ≥ 180 mm Hg (1 mm Hg = 0.133 kPa) or DBP ≥ 110 mm Hg after control.

This study was approved by the Ethics Committee of the Northern Jiangsu People's Hospital, and informed consent was obtained from all participating patients in this study.

### 2.2 Patient characteristics

The following data were collected: age, sex, cerebrovascular risk factors, National Institutes of Health Stroke Scale (NIHSS) scores on admission, THRIVE scores on admission, posterior circulation Alberta Stroke Program Early CT (pc‐ASPECT) scores on admission, use of intravenous recombinant tissue‐type plasminogen activator (rt‐PA), time to reperfusion, recanalization status, and laboratory results. Hemorrhage transformation (HT) during hospitalization was recorded. The composition of the THRIVE score was age, initial stroke severity according to the NIHSS score, and the presence or absence of hypertension (HTN), diabetes mellitus (DM), or atrial fibrillation (AF). The THRIVE score assigns 1 point for age 60–79 years, 2 points for age ≥ 80 years, 2 points for NIHSS score 11–20, 4 points for NIHSS score ≥ 21, and 1 point each for HTN, DM, and AF (Flint, Gupta, et al., 2014). The pc‐ASPECT scores on CT were assessed by a neuroradiologist before EST.

### 2.3 Endovascular procedures

All endovascular therapy was performed by one interventional neuroradiologist using neurovascular intervention. Cerebral angiography and endovascular therapy were performed under conscious sedation. Stent‐based thrombectomy with a Solitaire stent was performed as the first‐line endovascular treatment. When stent‐based thrombectomy was unsuccessful, additional mechanical approaches, such as balloon dilatation or stenting angioplasty, were performed. All patients underwent CT scans 24 hr after endovascular treatment. Recanalization status was reported by using the Thrombolysis in Cerebral Infarction (TICI) score based on the final angiogram, and successful recanalization was defined as a TICI score of 2b or 3 (Zaidat et al., 2013).

### 2.4 Outcomes

The primary study outcomes were poor functional outcome (defined as having an mRS ≥ 3) and all‐cause mortality at 90 days. The secondary outcome was HT, which was defined as a scattered point, piece, or block of high‐density shadows in the low‐density area of the CT scan after treatment or as a short T1 signal scattered in the long T1 area of the MRI scan. If there was high density caused by suspicious contrast agent leakage, a reexamination was conducted within 24 hr to identify blood (Che et al., 2017).

### 2.5 Statistical analysis

Continuous variables are expressed as the mean ± standard deviation (*SD*) or median (interquartile range [IQR]), and they were compared using the analysis of independent Student's *t* test or Wilcoxon rank‐sum test. Categorical variables are expressed as frequencies (%) and were compared using the chi‐square test. Multivariable logistic regression was performed to determine the independent association between the factors and the clinical outcomes, including poor functional outcome, all‐cause mortality at 3 months, and HT. All the variables with *p* < .2 in the univariate analysis were included in the multivariable analyses, with the full model developed by successively removing all nonsignificant covariates until all those remaining were statistically significant (*p* < .05). In addition, we tested the discriminatory ability of the THRIVE score and NIHSS score to predict poor functional outcome, all‐cause mortality, and HT by calculating C‐statistics (areas under the receiver operating characteristic [ROC] curve). All statistical analyses were performed using SPSS Statistics version 22.0 (IBM Corp.).

## 3 RESULTS

### 3.1 Patient characteristics

A total of 68 patients with acute BAO who underwent EST using Solitaire AB were enrolled in the study. The mean age was 66.94 (±10.95) years, and 49 (72.1%) of the patients were male. The median NIHSS score was 35 (IQR, 22.0–36.0). The median pc‐ASPECT score was 9 (IQR, 8.0–10.0). The median THRIVE score was 6 (IQR, 4.0–7.0). There were 26 (38.2%) patients with favorable outcomes (mRS ≤ 2), and 42 (61.8%) patients showed poor outcomes (mRS ≥ 3) at 3 months. Compared with participants with good functional outcomes, those with poor functional outcomes were more likely to be older and to have had a more severe stroke (higher NIHSS score), and they tended to have a higher THRIVE score. There was no significant difference between the pc‐ASPECT score and long‐term prognosis. Patients with poor outcomes also had a higher rate of hypertension than patients with good outcomes (Table 1).

**Table 1:** Clinical characteristics of the study population

	Favorable outcome (mRS 0–2) *n* = 26	Poor outcome (mRS 3–6) *n* = 42	*p* Value
Age, years (mean ± *SD*)	62.96 ± 12.26	69.40 ± 9.38	.017
Male sex (*n*, %)	21 (80.8%)	28 (66.7%)	.208
Admission NIHSS score (median, IQR)	20 (9–35)	35 (28–38)	.001
Admission pc‐ASPECT score (median, IQR)	9 (8–10)	9 (7–10)	.269
Admission THRIVE score (median, IQR)	4 (3–5)	7 (6–7)	<.001
Hypertension (*n*, %)	14 (53.8%)	36 (85.7%)	.04
Diabetes mellitus (*n*, %)	5 (19.2%)	16 (38.1%)	.102
Previous stroke or TIA (*n*, %)	3 (11.5%)	8 (19.0%)	.632
Coronary artery disease (*n*, %)	6 (23.1%)	3 (7.1%)	.129
Atrial fibrillation (*n*, %)	4 (15.4%)	15 (35.7%)	.124
Drinking (*n*, %)	5 (19.2%)	14 (33.3%)	.208
Smoking (*n*, %)	10 (38.5%)	12 (28.6%)	.397
Fasting blood glucose (mmol/L; mean ± *SD*)	7.32 ± 2.20	7.82 ± 2.69	.427
TC (mmol/L; mean ± *SD*)	1.46 ± 0.59	1.71 ± 1.48	.413
TG (mmol/L; mean ± *SD*)	4.64 ± 1.35	4.59 ± 1.33	.874
LDL‐C (mmol/L; mean ± *SD*)	2.94 ± 0.87	2.88 ± 0.97	.804
HDL‐C (mmol/L; mean ± *SD*)	1.25 ± 0.50	1.16 ± 0.37	.370
Time to recanalization, minute (mean ± *SD*)	267.65 ± 128.20	262.31 ± 150.66	.881
Intravenous thrombolysis (*n*, %)	2 (7.7%)	9 (21.4%)	.248
Intubation (*n*, %)	5 (19.2%)	7 (16.7%)	.516

### 3.2 The THRIVE score and 3‐month poor outcomes and all‐cause mortality

There were 42 (61.8%) participants who experienced poor functional outcomes, and 25 (36.8%) patients died from all causes during the 3‐month follow‐up. The multivariable logistic analysis showed that the baseline THRIVE score was significantly associated with poor functional outcomes (OR = 5.89, 95% CI 2.28–14.91, *p* < .001; Table 2). Table 2 also shows that the baseline THRIVE score (OR = 2.40, 95% CI 1.32–4.34, *p* = .004) and smoking (OR = 0.55, 95% CI 0.31–0.98, *p* = .041) were significantly associated with all‐cause mortality at 90 days according to the multivariable logistic analysis.

**Table 2:** Logistic regression analysis of poor outcome and mortality at 3 months

	Regression coefficients (*β*)	Standard error (*SE*)	OR	95% CI		*p* Value
Poor functional outcomes (mRS, 3–6) at 3 months						
Coronary artery disease	−2.04	1.23	0.13	0.01	1.43	.096
Admission NIHSS score	0.022	0.05	1.02	0.93	1.12	.645
Admission THRIVE score	−1.72	0.48	0.18	0.07	0.46	<.001
Mortality at 3 months						
Admission NIHSS score	0.00	0.05	1.00	0.92	1.10	.999
Admission THRIVE score	0.87	0.30	2.40	1.32	4.34	.004
Smoking	−0.60	0.29	0.55	0.31	0.98	.041
Previous stroke or TIA	0.54	0.79	1.71	0.36	8.09	.497
Intravenous thrombolysis	1.42	0.88	4.14	0.73	23.34	.108

Abbreviations: CI, confidence interval; NIHSS, National Institutes of Health Stroke Scale; OR, odds ratio; THRIVE, Totaled Health Risks in Vascular Events; TIA, transient ischemic attack.

### 3.3 The THRIVE score and hemorrhage transformation

There were 21 (30.9%) participants who experienced HT during the follow‐up. The multivariable logistic analysis indicated no significant association between the baseline NIHSS score (*p* = .559) or the THRIVE score (*p* = .607) and HT (Table 3).

**Table 3:** Logistic regression analysis of intracranial hemorrhage

	Regression coefficients (*β*)	Standard error (*SE*)	OR	95% CI		*p* Value
Admission NIHSS score	0.03	0.04	1.03	0.94	1.11	.559
Admission THRIVE score	0.14	0.27	1.15	0.68	1.94	.607
Male sex	1.59	0.89	4.92	0.86	28.25	.074
Coronary artery disease	−20.48	12,673.73	0.00	0.00	–	.999
Drinking	0.82	0.66	2.27	0.62	8.29	.216

Abbreviations: CI, confidence interval; NIHSS, National Institutes of Health Stroke Scale; OR, odds ratio; THRIVE, Totaled Health Risks in Vascular Events.

### 3.4 Receiver operator characteristic curve analysis comparing the thrive score with the NIHSS score

**Figure 1:**
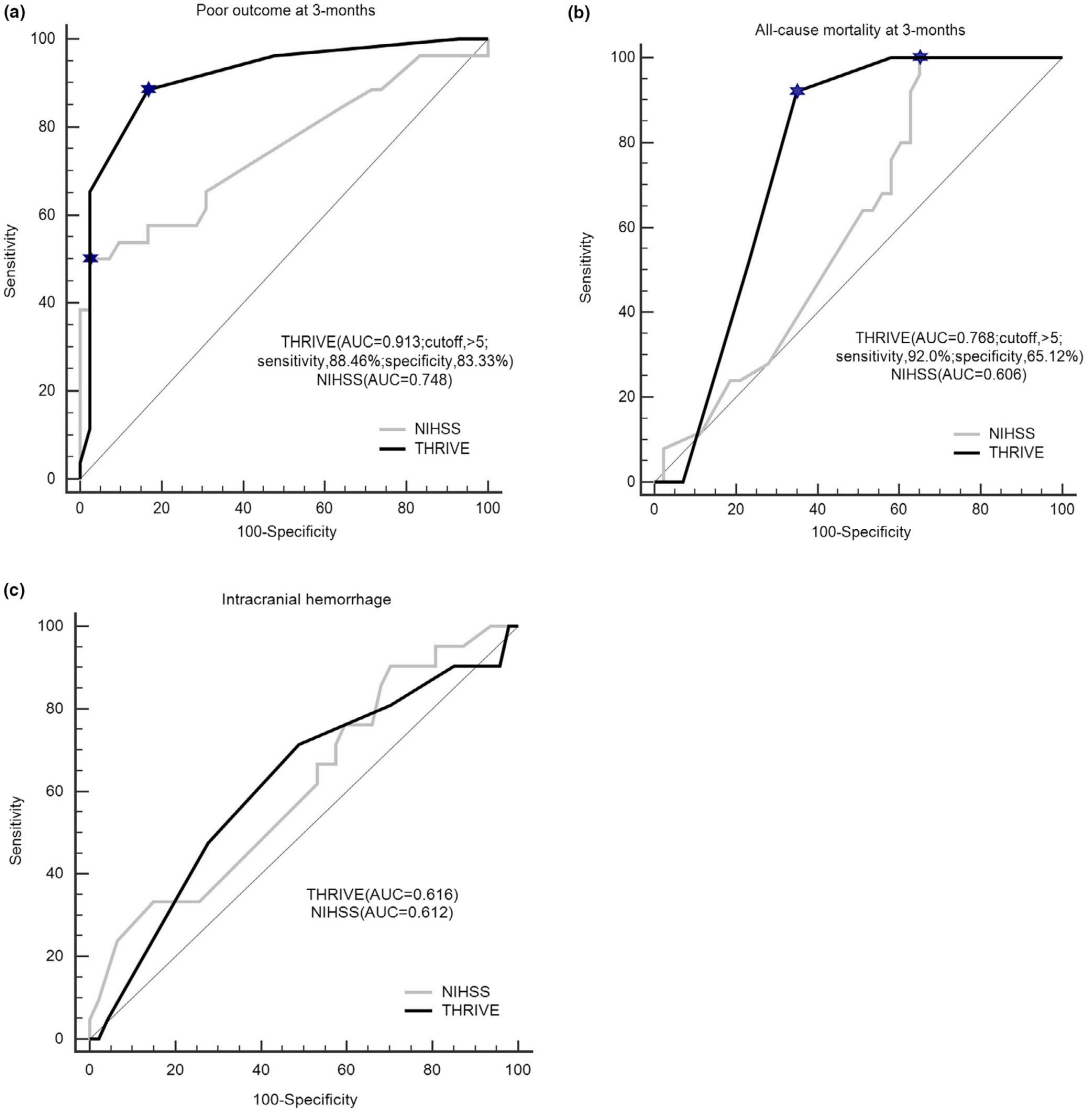
Comparison of the THRIVE score to the NIHSS score by ROC curve analysis. (a) ROC curve analysis for the predictive value of poor outcome at 3 months. (b) ROC curve analysis for the predictive value of all‐cause mortality at 3 months. (c) ROC curve analysis for the predictive value of intracranial hemorrhage

We used ROC curve analysis to compare the THRIVE score with the NIHSS score. Figure 1a,b shows that the ROC curves for the THRIVE score are superior to those for the NIHSS score in predicting poor functional outcome at 3 months and in predicting all‐cause mortality. For the prediction of poor functional outcome at 3 months, the AUC for the THRIVE score was 0.913 (cutoff > 5; sensitivity, 88.5%; specificity, 83.3%) compared with 0.748 for the NIHSS score (*p* = .0069). For the prediction of all‐cause mortality at 90 days, the AUC for the THRIVE score was 0.768 (cutoff > 5; sensitivity, 92.0%; specificity, 65.1%) compared with 0.606 for the NIHSS score (*p* = .0175). The THRIVE score had a similar AUC compared with the NIHSS score for the prediction of HT (AUC = 0.616 vs. AUC = 0.612, *p* = .957; Figure 1c).

## 4 DISCUSSION

The present study indicated that the THRIVE score performed well in predicting 90‐day clinical outcomes in BAO patients after endovascular treatment with a Solitaire AB stent. Patients with high THRIVE scores were more likely to have poor clinical outcomes at 3 months. The predictive value of the THRIVE score for outcome in BAO was superior to that of the NIHSS score. Thus, the THRIVE score is a suitable tool to predict prognosis in BAO patients who undergo EST.

The incidence of acute BAO is not high: approximately three percent of all stroke patients (Mattle, Arnold, & Lindsberg, 2011). Due to the different degrees of brainstem injury, patients can present with symptoms ranging from hemiplegia to coma. Although stroke treatment technology is advancing, BAO still has a high fatality rate, which reaches approximately 80%–90% without active intervention (Sarraj et al., 2015). In recent years, stent thrombectomy has been widely used in the acute stage of cerebrovascular occlusion. However, the evaluation and prognosis prediction of patients with this treatment still need to be elucidated. Although many patients with BAO fail to achieve an ideal prognosis after treatment with stent thrombolysis, there is still no superior means to achieve a favorable prognosis other than recanalization (Dornak, Herzig, & Sanak, 2014). The NIHSS score is the most commonly used tool to evaluate patients' symptoms and prognosis in stroke. However, the NHISS score for BAO is generally high, which is not conducive to the prediction of symptoms and prognosis in patients. In our study, we also evaluated the pc‐ASPECT score in each BAO patient, and the results showed that there was no significant difference in terms of long‐term prognosis.

The THRIVE score combines a patient's functional assessment with risk factors such as old age, hypertension, and hyperglycemia, which are closely related to prognosis in ischemic stroke (Nakayama et al., 1994; Steger et al., 2004). A number of clinical reviews have shown that the THRIVE score has been validated across different acute stroke treatments, including no treatment, intravenous thrombolysis (IVT), and endovascular stent treatment (Flint et al., 2010; Kamel et al., 2013). In many EST trials, the THRIVE score was developed using patients from the MERCI and Multi‐MERCI trials (Flint et al., 2010). Then, in the TREVO‐2, SWIFT, and STAR trials, the THRIVE score also strongly predicted long‐term prognosis in the patients after EST. A. Kastrup's team found that the THRIVE score predicted outcome after endovascular therapy in patients with anterior circulation stroke (Kastrup et al., 2017). However, data on the association between the THRIVE score and prognosis in AIS patients with BAO who underwent EST treatment were limited. Previous studies have confirmed that the status of vascular recanalization directly affects patient prognosis after EST. The success rate of recanalization is affected by many factors, such as the patient's vascular condition, the doctor's technical level, and medical conditions (Gory et al., 2018; Papanagiotou et al., 2018). Therefore, we selected patients with good recanalization (TICI 2b or 3) in our study. As a novel finding, our data demonstrated that the THRIVE score could strongly predict prognosis in BAO patients after EST with good recanalization. The THRIVE score was an independent predictor of poor prognosis and mortality at 90 days in the patients. Neither the THRIVE score nor the NHISS score were clearly associated with HT after EST in BAO patients. Similar conclusions have been shown in previous studies in patients with anterior circulation stroke (Flint, Cullen, et al., 2014).

The NHISS score has always been the most important tool for the assessment of ischemic stroke. A large number of studies show that the NIHSS score is a good predictor of prognosis in stroke patients (Dornak et al., 2014). Wang's study found that the THRIVE score was a better predictor than the NIHSS score in Chinese patients after thrombolysis (Chen et al., 2015). To further determine the predictive value of the THRIVE score for such patients, we used an ROC curve to compare the predictive value of the THRIVE score with that of the NHISS score and to explore the THRIVE cutoff point for poor prognosis and mortality. Our data showed that the predictive value of the THRIVE score for poor prognosis and mortality at 90 days was superior to that of the NIHSS score. Our data also showed that a patient with a THRIVE score equal to or greater than 6 was significantly associated with poor functional outcome and mortality at 90 days.

The strengths of this study are that it was the first to evaluate the predictive value of the THRIVE score for 90‐day mortality and patient outcome in AIS patients with BAO who underwent EST treatment. However, there are still some potential limitations. First, the data were from a single clinical center, and the study was limited by the small sample size. Moreover, we did not analyze the cause of all‐cause mortality due to the severity of the disease, as the local custom is to exclude autopsies. Finally, data on the administration of prestroke medicines, which may influence the clinical outcome, were not collected.

In summary, the THRIVE score was found to be an independent predictor of clinical outcome in BAO patients who underwent EST. Therefore, the THRIVE score can quickly predict functional outcome and mortality before EST. This tool will assist clinicians in choosing treatment methods and in evaluating the prognosis of these patients.

## CONFLICT OF INTEREST

No potential conflict of interest was reported by the authors.

## Data Availability

The data that support the findings of this study are openly available in additional material.
